# Re-defininG AddiC^CH3^Tion: genomics and epigenomics on substance use disorders

**DOI:** 10.1002/mgg3.93

**Published:** 2014-07-10

**Authors:** Joni L Rutter, Nora D Volkow

**Affiliations:** National Institute on Drug Abuse, National Institutes of HealthBethesda, Maryland, 20892

Drug addiction is a term applied across substance use disorders (SUDs) and defined as a chronic, relapsing complex brain disease characterized by compulsive drug seeking, craving, loss of self-control, and impaired decision making (National Institute on Drug Abuse 2010). Drug addiction persists in spite of harmful consequences and cuts across all demographics. Addiction results from the interactive effects among multiple genes, acting in multiple environments, and across multiple stages of development. It is this intersection that roots SUDs, in concert with other biological processes, in genetic and epigenetic mechanisms that provide a scaffold for normal brain development, for learning and memory, and for pathophysiology.

The heritability of addictions ranges from moderate to high. Twin studies have shown that genetic factors account for approximately half of the variance for addictive disorders (Kendler et al. 2008). It is thought that the remaining heritability comes from environmental and developmental aspects, including epigenetic factors that contribute to gene expression miscues of otherwise highly synchronized gene regulation. Over the last decade, genetic research on SUDs has transitioned from candidate gene and linkage methods to genome-wide association studies (GWAS) and sequencing-based approaches. Many studies have been fruitful mainly because studying the genetics of SUDs benefits from a vast knowledge of a given drug's mechanism of action (Rutter 2006). Among these are genes encoding for the enzymes involved with drug metabolism such as alcohol and aldehyde dehydrogenases, which offer protection against alcoholism (Chen et al. 2009) and CYP2A6, which offers protections against nicotine addiction (Malaiyandi et al. 2005). Studies have also identified gene variants that modulate the response to the rewarding or aversive effects of drugs such as the nicotinic cholinergic receptor subunit gene (*CHRNA5*, *α5*) for nicotine (Fowler et al. 2011) and the *μ*-opioid receptor gene (*ORPM1*) for alcohol and opioids (Arias et al. 2006). Finally genes have been identified that may help predict outcomes for naltrexone treatment for alcohol dependence, such as the A118G *OPRM1* single-nucleotide polymorphism (SNP) (Enoch 2013) and for response to nicotine replacement therapy for smoking cessation, such as the D398N SNP in the *α5* gene and CYP2A6 genetic polymorphisms (Chen et al. 2014a,b).

As with many complex disorders, clear GWAS successes have been hard to come by. However, a striking example in the field of nicotine dependence is the *α5*-*α3*-*β4* nicotinic cholinergic receptor subunit gene cluster on chromosome 15q, which sparked the discovery of several loci related to smoking and its more dire health consequences, such as lung diseases and peripheral artery disease (Volkow et al. 2008a; Welter et al. 2014). While found initially via a hybrid candidate gene-GWAS design (Saccone et al. 2007), the *α5*-*α3*-*β4* gene cluster was replicated by GWAS and meta-analysis approaches to become one of the most replicated variants in complex disease genetics (Liu et al.^2010^; Thorgeirsson et al. 2010; Tobacco and Genetics Consortium 2010). This seminal finding pointed to lesser known targets for nicotine addiction (the *α*5-*α*3-*β*4 nicotine subunit receptors) in addition to the more common *α*4/*β*2 nicotinic receptors, which are thought to mediate nicotine reward and dependence (Picciotto et al. 1998).

This GWAS finding was also crucial for identifying the importance of the medial habenula (MHb) and the interpeduncular nucleus (IPN), brain regions that express high levels of the *α5* subunit containing receptors (Changeux 2010), and which form a brain circuit that plays a role in the aversive effects of nicotine. Since the MHb-IPN inhibits the activity of brain dopamine neurons involved with reward, the *α5* gene pointed to the importance of the aversive effects of nicotine in the process of addiction. Indeed *α5* knockout mice are much less sensitive to the aversive effects of high nicotine doses than are wild-type mice which avoid high nicotine doses (Fowler et al. 2011). Combined with the dense expression of *α*5 in the MHb-IPN, a picture emerged which implicates nicotinic receptors containing *α5* as a “gate” for nicotine intake. When the *α5* gene contains the common SNP the gate is intact and the MHb-IPN aversive circuit functions properly. However, when the *α5* gene contains the variant SNP, the gate is weakened and the aversive protection is reduced (Fowler et al. 2011; Fowler and Kenny 2012). This could explain why the heaviest smokers are more likely to have the associated risk allele.

In parallel to these large-scale genomic studies, significant advances in neuroimaging have led to the recognition of the crucial role that the orchestration of gene regulation plays at specific developmental stages in the development of the human brain. This has also led to an increased recognition that the regulation of genes that modulate vulnerability (or resilience) for SUD is mediated though their influence on brain development, connectivity, and function, which in turn influences brain responses. This more integrated perspective on the role of genes and gene regulation in SUD provides a better understanding of why there is significant comorbidity between SUD and mental illness, including not only the frequent co-occurrence of both of these disorders but also overlap in brain circuits, in risk alleles and in environmental insults. Particularly prominent are circuits involved with “self regulation and control,” which implicate frontal networks and those involved with saliency circuits, which implicate striatal and limbic regions (Volkow et al. 2008b). For example, early social stressful exposure during childhood, the sensitivity of which is modulated by the gene that encodes for the serotonin transporter (Drabant et al. 2012) increases the risk of depression and SUD and is associated with disrupted frontal control networks. Another example is the 7-repeat variable number tandem repeat (VNTR) in the *DRD4* gene with individuals showing a greater risk for attention deficit hyperactivity disorder (ADHD) and for SUD. These individuals also show an increased sensitivity to both adverse as well as positive environmental factors resulting in either worse or better outcomes than those without the 7-repeat *DRD4* VNTR (Grady et al. 2013; Olsson et al. 2013). This is interpreted to reflect the *DRD4* role in modulating dopamine signaling in frontal striatal terminals (Nikolova et al. 2011).

Outside of the large-scale studies, the molecular genetic contributions to SUD indicate a staged effect on the transitions that uncover points of entry into addiction. These include genes that modulate early experimentation with drugs such as those involved with personality (Belcher et al. 2014); genes that determine sensitivity to rewarding versus aversive effects of drugs (Shabani et al. 2011); genes that modulate conditioning to the drug, which implicate genes involved with neuronal plasticity and learning (Drgon et al. 2010); genes that involve the transition into compulsive drug intake which implicate genes involved with regulation of self-control networks (Gustavson et al. 2014); genes that mediate the sensitivity to symptoms of drug withdrawal and likely involved in the regulation of the amygdala and the habenula and other networks involved in stress reactivity and dysphoria (Wankerl et al. 2010); genes involved in the regulation of interoceptive awareness such as the insula and the default mode network (Vergara et al. 2014); and finally genes that regulate the response to treatment (Gonzalez et al. 2012). A prospective longitudinal study points out that much of the genetic variants that regulate the growth and development of the human brain have been important for the evolution of the cerebral cortex (Schmitt et al. 2014). Indeed, some of these genes are involved in the very basic neural-synaptic activities that differentiate the patterned expression in mouse and humans (Miller et al. 2014).

By the same token, given that drug addiction is comorbid with many psychiatric conditions such as depression, anxiety, ADHD, and schizophrenia (Buckley et al. 2009), it is difficult to interpret whether the drug addiction is a cause or a consequence of that comorbidity. One possibility is that these distinct clinical disorders have common biological underpinnings. A case in point is a study that found that SNPs associated with euphoric responses to d-amphetamine also showed decreased susceptibility to schizophrenia and ADHD and suggest a point of convergence of SNPs within the dopamine system genes affecting multiple disorders (Hart et al. 2014). Furthermore, marijuana, which is the most frequently used illicit substance and is more commonly used during early adolescence, has been associated with twofold increased risk of schizophrenia (Andreasson et al. 1987; Arseneault et al. 2002). Recognizing that most schizophrenia patients have no prior marijuana use, and most adolescents who abuse marijuana do not develop schizophrenia, the link spurred investigations into the impact of genetic variation in the cannabinoid receptor 1 (*CNR1*). CNR1 is densely localized in the hippocampus, the amygdala, and the prefrontal cortex (Herkenham et al. 1991) and is the primary receptor for Δ9-tetrahydrocannabinol (THC), the psychoactive component in marijuana. Reduced hippocampal volumes are more likely seen in heavy marijuana users relative to healthy controls (Ashtari et al. 2011), and this volume reduction is mediated by a common variant in *CNR1* (Schact et al. 2012). Furthermore, several reports have found that polymorphisms within *CNR1* are associated with white matter brain volumes, which might represent a gene × environment relationship for the brain volume deficits in schizophrenia (Ho et al. 2011).

The high prevalence of cigarette smoking among schizophrenics also reinforces the hypothesis that addictive behavior and schizophrenia may rely on shared neurocircuitry. For instance, ∼90% of people with schizophrenia smoke and use nicotine as a mechanism of self-medication to increase cognition (Chambers et al. 2001). Schizophrenics may have underlying genetic differences in regions of the brain that are similar to those seen in long-term substance users but without the prior drug exposure. For example, studies of resting state functional connectivity (rsFC) in schizophrenia patients found that they had reduced circuit strength in the dorsal anterior cingulate cortex (dACC) and ventral striatum regardless of smoking status, whereas control heavy smokers had reduced connectivity in this pathway compared to control nonsmokers (Moran et al. 2012). These imaging data suggest that schizophrenia patients may be vulnerable to smoking due to these dACC circuit weaknesses. In turn the D398N *α5* gene variant is associated with strength of connectivity in the dACC-ventral striatum/extended amygdala circuit, such that the risk allele associates with decreased rsFC (Hong et al. 2010).

In addition to the direct role of genetics in SUD and related comorbidities, genetic variation also plays a role in the more subtle traits that are commonly linked with addiction. Personality traits, for example, are influenced by a myriad of factors. Genes and circuits involved with novelty seeking, impulsivity, low reward sensitivity, and conduct disorder or antisocial personality disorder are generally thought to be unifying traits in SUDs – that is, they are largely associated with early-life behaviors that tend to manifest as later-life problems with SUDs. However, there seems to be a high degree of disease heterogeneity that is dependent on how and when those early traits develop in the context of environmental stressors, such as maltreatment and abuse, as well as the genetic vulnerabilities that might be at play (Dick et al. 2013). Examples of such genes include gamma-aminobutyric acid receptor alpha 2, serotonin transporter 5-HTT, and monoamine oxidase A (Caspi et al. 2002; Dick et al. 2013; Ernst et al. 2014). It is not surprising that genetic variants within these neurotransmitter genes can converge to impact mood, aggression, and impulsivity, and to impact later-life addiction vulnerability.

Similarly, genes that modulate homeostatic control may help counteract the risk of compulsive drug use, or overeating in food-rich environments. The *CNR1* gene is a good example of a gene involved in both reward and homeostatic pathways. It has been associated with body mass index (BMI) and obesity risk (Schleinitz et al. 2010), as well as with addiction (Benyamina et al. 2010). Similarly endogenous opioids are involved in hedonic responses to food and to drugs, and the functional A118G polymorphism in the *OPRM1* has been associated with vulnerability for binge eating disorders (Davis et al. 2009) and for alcoholism (Ray et al. 2011). A more recent study used GWAS and meta-analysis approaches to examine the observation that smoking influences weight gain, a common reason for relapse especially for women. The *α5*/*α3*/*β4* cluster most known for its role in nicotine dependence is also associated with BMI where the risk allele for nicotine dependence correlates with reduced BMI in current and former smokers, but not never-smokers (Freathy et al. 2011). Another study approached the question by examining common variants identified in a GWAS of BMI and compared smokers to never-smokers and found that transmembrane protein 18, mitochondrial translational initiation factor 3, and brain-derived neurotrophic factor (BDNF) are also associated with smoking initiation and/or cigarettes per day (Thorgeirsson et al. 2013). Taken together, these data support the notion that phenotypic outcomes such as obesity, nicotine dependence, alcoholism, *inter alia* are a complex manifestation of multiple genetic variants acting within a common biological network.

By the same token, genetic differences in personality and emotion influence an individual's stress reactivity, which has implications on drug use vulnerability. The opioid and cholinergic systems provide classic examples that link the stress systems to SUDs. Responses to stress invoke very rapid and robust brain responses through the opioid, norepinephrine (NE), orexin, and corticotropin-releasing factor (CRF) systems and they can remain engaged even when the stressor is no longer present (Koob 1999). The CRF system is highly responsive to the environment and genetic studies support the role of CRF serving as a key interface between environmental stressors and an individual's vulnerability to stress-related psychiatric disorders (Binder and Nemeroff 2010). Sensitivity to stress has a propensity to associate with sensitivity to pain, where the opioid system is involved. A study using rats that were selectively bred for high responding to emotional reactivity and exploratory behavior showed that in the high responding rats, the metabotropic glutamate receptors were found to modulate neuronal excitability and plasticity most notably in the hippocampus during neurodevelopment and synaptogenesis (Clinton et al. 2011), illustrating a caricature of the stress/anxiety systems that intersect with the vulnerability to addiction.

It has become increasingly evident that epigenetic factors are instrumental in their ability to affect several genes simultaneously with or without the underlying genetic variation. Epigenomic regulation mediates cellular responses to external stimuli such as environmental toxins, infection, stress, and drugs of abuse. Epigenomic regulation of cocaine responses in brain reward circuitry has been particularly well studied (Nester 2014). Exposure to drugs of abuse also may have epigenetic transgenerational influences that affect offspring (Vassoler and Sadri-Vakili 2014). For example, male rat offspring sired by cocaine exposed males were slower to acquire cocaine self-administration than were offspring sired by unexposed males and the effects were linked to increased acetylation of the promoter region of the BDNF gene (Vassoler et al. 2013). A recent study of THC exposure during adolescence found that the unexposed offspring showed an increased propensity to self-administer heroin (Szutorisz et al. 2014). Taken together, these examples indicate the pervasive influence of environmental factors, which are mediated through epigenetic mechanisms; and that these influences may not only affect cellular events in the exposed, but may also manifest across generations.

**Figure 1:**
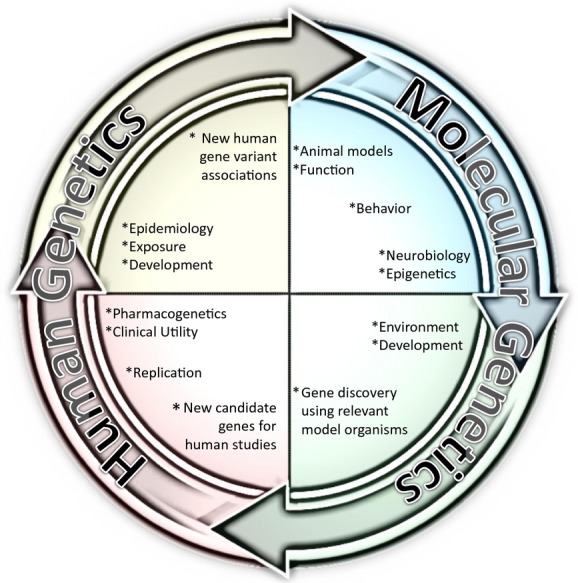
Schematic of the genetics of addiction research cycle. Discovery of genetic and epigenetic processes important in addiction requires an integrated approach, involving genetic and epigenetic factors, and their functional relevance at the molecular, cellular, circuit, and behavioral level. The iterative cycle benefits from both human genetics and molecular genetics to address each step of identification, replication, functional characterization, causality determination, and potential clinical implications.

In summary, genetics and epigenetics have expanded our understanding of the neurobiological basis of SUDs and related disorders through an interdisciplinary process of human and molecular genetic research (Fig. 1). The remarkable success using genomics, especially in the field of nicotine dependence and smoking cessation, is now starting to enter the realm of genomic medicine (Bergen et al. 2013; Chen et al. 2014a,b). Treatment approaches that capitalize on genomic information about an individual's genetic or metabolic profile signal a pharmacogenetic game changer in choosing medical and treatment decisions. Likewise, this work weaves a broader perspective on the role of genes, their expression, and on the effects of environmental exposures and experiences on behavior. New approaches such as the CRISPR/Cas9 and TALEN gene editing systems, used for silencing, enhancing, or changing specific genes, will be important tools to allow more specific exploration of how genes associated with SUD influence behavior in animal models of addiction. Research toward understanding the spatial and temporal aspects of the precise molecular mechanisms and pathways influenced by genes at the cellular and circuit levels is beginning to reveal that addiction is a disease of the developing brain influenced by genetic and epigenetic mechanisms. A more comprehensive genomic and epigenomic understanding of SUDs will guide future efforts toward a new way of thinking about treatments and prevention.
